# Motor Cortex Plasticity during Unilateral Finger Movement with Mirror Visual Feedback

**DOI:** 10.1155/2016/6087896

**Published:** 2015-12-31

**Authors:** Hatice Kumru, Sergiu Albu, Raul Pelayo, John Rothwell, Eloy Opisso, Daniel Leon, Dolor Soler, Josep Maria Tormos

**Affiliations:** ^1^Fundación Institut Guttmann, Institut Universitari de Neurorehabilitació adscrit a la UAB, Badalona, 08916 Barcelona, Spain; ^2^Universidad Autonoma de Barcelona, Bellaterra, 08193 Cerdanyola del Vallès, Spain; ^3^Fundació Institut d'Investigació en Ciències de la Salut Germans Trias i Pujol, Badalona, Barcelona, Spain; ^4^Texas A&M University, College Station, TX 77843-4235, USA; ^5^Institute of Neurology, University College London, London WC1N 3BG, UK

## Abstract

Plasticity is one of the most important physiological mechanisms underlying motor recovery from brain lesions. Rehabilitation methods, such as mirror visual feedback therapy, which are based on multisensory integration of motor, cognitive, and perceptual processes, are considered effective methods to induce cortical reorganization. The present study investigated 3 different types of visual feedback (direct, mirrored, and blocked visual feedback: DVF, MVF, and BVF, resp.) on M1 cortex excitability and intracortical inhibition/facilitation at rest and during phasic unimanual motor task in 11 healthy individuals. The excitability of the ipsilateral M1 cortex and the intracortical facilitation increased during motor task performance in the DVF and MVF but not in the BVF condition. In addition, MVF induced cortical disinhibition of the ipsilateral hemisphere to the index finger performing the motor task, which was greater when compared to the BVF and restricted to the homologue first dorsal interosseous muscle. The visual feedback is relevant to M1 cortex excitability modulation but the MVF plays a crucial role in promoting changes in intracortical inhibition in comparison to BVF. Altogether, it can be concluded that a combination of motor training with MVF therapy may induce more robust neuroplastic changes through multisensory integration that is relevant to motor rehabilitation.

## 1. Introduction

Change of balance in cortical and intracortical excitability is one of the most important neurophysiological mechanisms underlying motor recovery from brain lesions such as a stroke [1]. Motor recovery after an unilateral stroke depends on plasticity as an intrinsic property of the nervous system that can result in adaptive or maladaptive consequences, which includes dynamic interhemispheric competition through excitatory/inhibitory mechanisms between the unaffected and affected hemisphere [2]. Methods based on multisensory integration of motor, cognitive, and perceptual processes through action observation, mental training, and virtual reality have been proven to be effective methods to induce more efficient cortical reorganization and to promote functional recovery in stroke patients [3, 4].

Passive movement observation from a first person perspective, in absence of overt movement of either limb, facilitates M1 excitability [5–7] through activation of the same motor pathways that are recruited in observers when actually performing the observed movement [8]. Mirror visual feedback (MVF) therapy, which represents the illusory perception of the movements of the active limb as movements of the inactive limb, is a more complex method involving visual and kinesthetic feedback during observation and action execution [9]. Compared to passive movement observation, MVF was associated with enhanced engagement of the M1 cortex controlling the active hand and also induced additional activation in the contralateral M1, the supplementary motor area, the supramarginal gyrus, the superior parietal lobe, and the primary and higher-order visual areas involved in solving the perceptual incongruences [10–12]. Most studies [5, 6, 13] have focused mainly on the assessment of the changes at corticospinal excitability induced by motor tasks, while it remains unknown whether the plastic changes are widespread or localized to the M1 cortical area controlling the muscles responsible for the motor task.

Our study evaluates the effects of 3 different types of visual feedback (direct, mirror, and blocked visual feedback) on ipsilateral motor cortex excitability and the ipsilateral motor cortex inhibition/facilitation during unimanual motor task. The same measurements were taken from an adjacent muscle not involved in the motor task to evaluate whether the plastic changes of ipsilateral M1 area are widespread or circumscribed to the cortical area controlling the unilateral movement.

## 2. Methods

Eleven healthy subjects (4 men, 7 women; 9 right-handed, according to the Edinburgh handedness inventory (EHI) [14]) participated in the study (EHI score: 83.3 ± 14.1 for right-handed participants; −75.0 ± 7.1 for left handed participants). Subjects with known neurological disorders or symptoms suggestive of central or peripheral neurological diseases or contraindications for Transcranial Magnetic Stimulation (TMS) have not been included in the study. All procedures were approved by the local Research Ethics Committee of the Institut Guttmann and all participants signed written informed consent.

### 2.1. EMG Recordings

Surface EMG was recorded from the first dorsal interosseous (FDI) muscle and also from the abductor digiti minimi (ADM) muscles of the nondominant (inactive) hand using silver/silver chloride (Ag/AgCl) disc electrodes with an outer diameter of 0.9 cm, prior skin preparation by rinsing and degreasing. The EMG signal was amplified using a conventional EMG machine (Medelec Synergy, Oxford Instruments; Surrey, England) using a band-pass of 50 Hz–1 kHz and a sensitivity of 0.5 mV per division. Sweep duration was 100 milliseconds. The recordings were stored into a Synergy computer for offline analysis.

#### 2.1.1. Transcranial Magnetic Stimulation (TMS)

TMS was generated by a Magstim 200 stimulator (The Magstim Company, Dyfed, UK) and delivered through a figure-of-eight coil (outer diameter of each wing 8 cm). Participants were wearing a swimming cap to mark the hot spot. The coil was placed on the scalp over the hand motor area of the nondominant hemisphere for eliciting MEPs in the FDI muscle and the EMG activity in the ADM muscle was simultaneously recorded. The optimal scalp position for eliciting MEPs in the FDI muscle of the nondominant (inactive) was determined as the area from which suprathreshold stimuli elicited maximal amplitude MEPs. The coil was held manually with the intersection of the coil placed tangentially to the surface of the scalp and the handle pointing backward and laterally at an angle of 45° to the sagittal plane, which is considered the optimal position to generate a posterior-to-anterior current flow in the brain. The optimal position was marked on the white swimming cap with a red pen to ensure a constant location throughout the experiments. During the experiments we checked the location of the coil over the hot spot after each of 7-8 stimuli for consistency.

First we determined the resting motor threshold (RMT), which was defined as the minimum stimulation intensity that produced an MEP in the FDI muscle of the nondominant hand with peak-to-peak amplitude greater than 50 *μ*V in at least 5 of 10 consecutive trials. Single pulse TMS (spTMS) at suprathreshold intensity (120% of the RMT) was used to elicit MEPs in the FDI of the nondominant (inactive) hand. We also evaluated the short intracortical inhibition (SICI) and short intracortical facilitation (SICF) using paired-pulse TMS (ppTMS) [15] by applying two stimuli: a subthreshold conditioning stimulus (80% of RMT) and a suprathreshold test stimulus (120% of RMT) at interstimulus interval (ISI) of 2 ms for SICI and 10 ms for SICF. Each assessment included 16 trials at each ISI.

### 2.2. Procedure

The subjects were seated in a chair with their forearms and hands resting in neutral positions on the table in front of them. All participants underwent 3 different experiments: direct visual feedback (DVF), mirror visual feedback (MVF), and blocked visual feedback (BVF) with 2 different conditions: both hands at rest and during unimanual motor task.

During the resting condition the subjects were instructed not to move their hands and to keep focusing their attention on the dominant hand. During the motor task, the participants were asked to perform sequential unilateral movements consisting in touching with the index finger of the dominant hand a 2 cm dot on the table, which was 5 cm away from the dominant index finger, at a frequency of 2-3 Hz. The participants were given 5–10 minutes to practice the FDI movement.

**Experiment 1 .**

DVF from the hands at rest versus motor task with the dominant hand: although in this experiment participants could see both hands on the table, they were asked to attend the dominant hand.

**Experiment 2 .**

**Figure 1:**
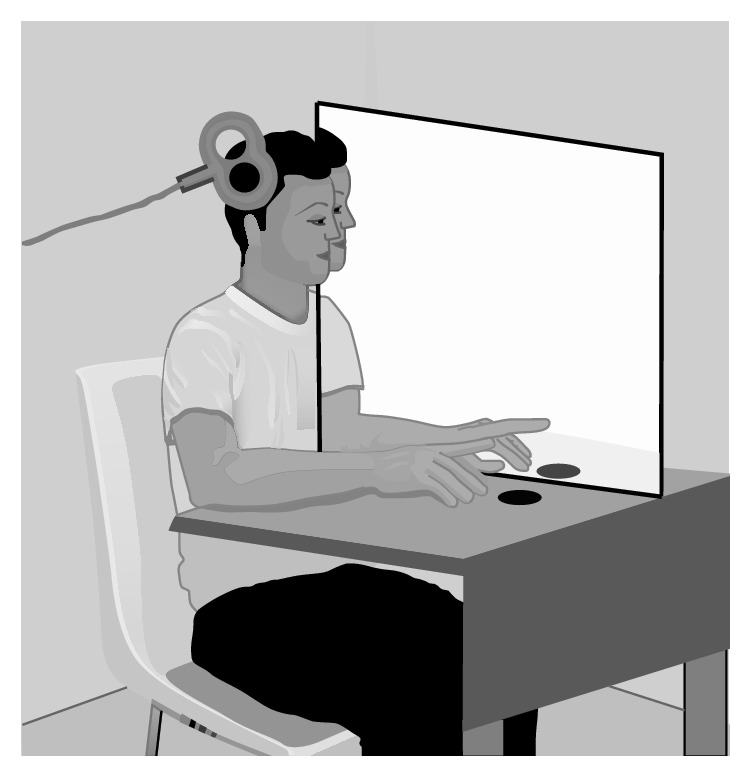
Schematic representation of the mirror visual feedback condition during motor task.

MVF from the hands at rest versus motor task with the dominant hand: for this experiment a mirror was placed vertically in the midsagittal plane in front of subjects such that they could see the mirror reflection of their active hand, which appeared superimposed on top of the unseen inactive hand. The participants were asked to attend the mirror-reflection of their dominant hand at rest and during movement performance (Figure 1).

**Experiment 3 .**

BFV from the hands at rest versus motor task with the dominant hand: during this experiment the mirror was replaced with an opaque block so that participants could not see any reflection of the dominant hand at rest, neither during motor task. The subjects were instructed to look only at the opaque block at rest and during unilateral movement task (without seeing the dominant “active” hand, neither nondominant “inactive” hand). Confirmation that the dominant hand was not visible was assessed by verbally asking the subjects.

All subjects performed the experiments in the same order as they are listed above: DFV, MVF, and BVF.

In each experimental condition we recorded MEP, SICI, and SCIF as follows: 16 MEPs elicited by spTMS, 16 MEPs evoked by ppTMS to assess SICI, and 16 MEPs elicited by ppTMS to evaluate SICF at rest and during unilateral movement task. All experiments were performed on the same day with 10–15 minutes break between experiments. Between rest and movement conditions in each experiment, the participants were given a 5–10-minute training of finger-tapping. The approximate duration of the study was 2.5 hours.

EMG activity of the FDI and ADM muscles in the no-dominant (inactive) hand was carefully monitored online to ensure that relaxation was maintained. Individual MEPs were excluded from the analysis and the trial was repeated if the EMG activity in the inactive FDI during the 50 ms immediately preceding the TMS pulse exceeded 50 *μ*V of amplitude. Overall, between 0 and 4 MEP recordings per participant were rejected in each experimental condition for different reasons, for example, the participants had difficulties maintaining relaxation of the target FDI muscle, or because of erroneous delivery of spTMS instead of ppTMS, and so forth (mean ± standard deviation of rejected MEP recordings in the resting condition for all participants was 1.1 ± 1.0 and of rejected MEP recordings during motor task performance was 1.9 ± 1.2).

### 2.3. Data and Statistical Analysis

We measured the peak-to-peak amplitude of MEP (*μ*V) of the FDI and ADM muscles in each recording then we calculated the mean amplitude of MEPs for each individual and experimental condition. Changes in MEP amplitude in different experimental conditions were calculated as percentage changes in the mean amplitude of single-pulse or paired-pulse TMS-induced MEP compared with that of single-pulse TMS-induced MEPs at rest.

The statistical analysis was performed with a commercial software packages (SPSS, version 17.0, SPSS Inc., Chicago, IL, USA). Based on Shapiro-Wilk test most data in our study failed the normality assumption. Because log transformation and sqrt transformation failed to normalize the data we performed the Friedman test. The small sample size was an additional factor that was considered when running nonparametric analysis. We used the Friedman test with Wilcoxon's test as post hoc analysis to evaluate changes in MEP, SICI, and SICF between resting and motor task condition in the same experiment and then to compare percentage changes between 3 experimental conditions. The data are expressed as mean and standard deviation and the level of significance was set at *p* < 0.05 for all tests.

## 3. Results

### 3.1. Demographic Data and Baseline Corticospinal Excitability

**Figure 2:**
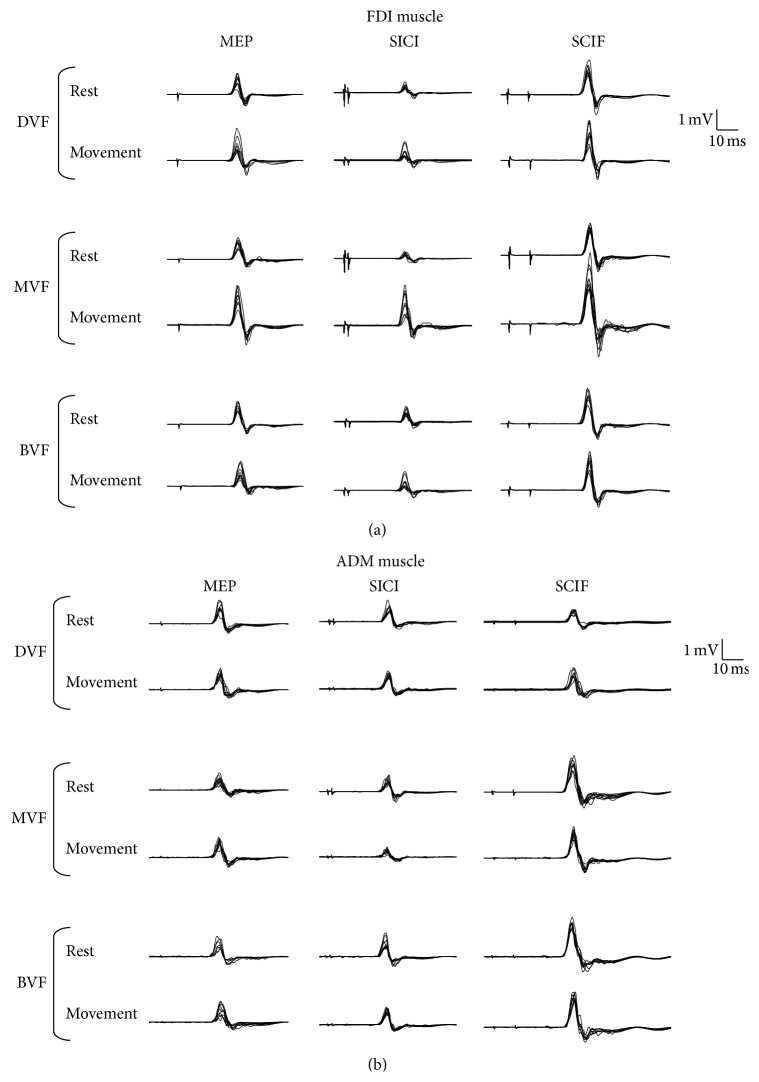
Representative MEPs using spTMs and SICI and SICF recorded in the nondominant FDI and ADM muscles in a 32-year-old healthy man at rest and during performance of motor task in different experimental conditions (DVF, MVF, and BVF).

The mean age was 29.6 ± 5.3 years with range: 21–41 years (Table 1).  Figure 2 shows representative MEPs using spTMs, and SICI and SICF recorded in the nondominant FDI and ADM muscles in a 32 year-old healthy man.

### 3.2. Corticospinal Excitability Modulation

The amplitude of MEPs in FDI and ADM muscles at rest did not differ between experiments (*χ* ^2^(2) = 0.73, *p* = 0.69, Friedman test) (Table 2). Seeing the active hand performing the motor task, either directly (DVF) or in the mirror (MVF), was associated with a statistically significant increase in the MEPs amplitude compared to resting condition in the DVF (*p* = 0.008, Wilcoxon test) and MVF (*p* = 0.01, Wilcoxon test) but not in the BVF condition (*p* = 0.42, Wilcoxon test).

### 3.3. Changes in Short Intracortical Inhibition and Facilitation

The SICI and SICF at rest did not differ between experiments (SICI, *χ* ^2^(2) = 0.6, *p* = 0.74; SICF, *χ* ^2^(2) = 1.3, *p* = 0.53; Friedman test).

Motor task performance with DVF increased the SICF (*p* = 0.03, Wilcoxon test) whereas SICI was not modulated (*p* = 0.29, Wilcoxon test). In the MVF experimental condition during the motor task, SICI was reduced (disinhibited) significantly (*p* = 0.003, Wilcoxon test) and SICF augmented also significantly (*p* = 0.013, Wilcoxon test). The motor task performance in the BVF condition did not change SICI or SICF (*p* > 0.3 for SICI and SCIF, Wilcoxon test for both comparisons).

The MEPs, SICI, and SICF of ADM (task unrelated) muscle did not change during the motor task performance with the FDI muscle (*p*s > 0.05).

### 3.4. Comparison between Experimental Conditions

**Figure 3:**
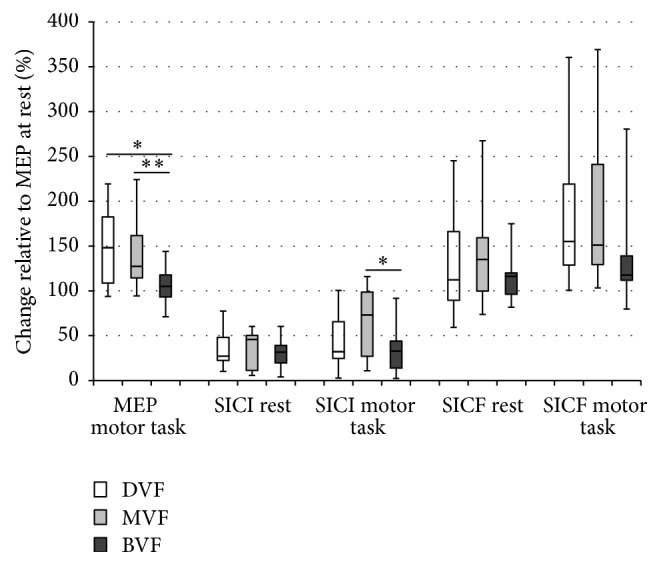
Percentage change (%) of the FDI MEP, SICI, and SICF at rest and during motor task with different visual feedback (DVF, MVF, and BVF).

The percentage of change (%) in MEPs amplitude elicited by spTMS in FDI during motor task was significant between experiment comparisons (*χ* ^2^(2) = 7.1, *p* = 0.03, Friedman test) with higher % changes of MEP amplitude in the DVF (*p* = 0.016, Wilcoxon test) and MVF (*p* = 0.004, Wilcoxon test) condition compared to BVF but not when comparing between MVF and DVF conditions (*p* = 0.6, Wilcoxon test). Furthermore, the motor task modulated significantly the % changes in SICI (*χ* ^2^(2) = 6.7, *p* = 0.035; Friedman test) (Figure 3). The % of change in SICI was significant across experiments, with stronger disinhibition in the MVF condition compared to BVF (*p* = 0.01, Wilcoxon test) but not when compared to DVF (*p* = 0.09, Wilcoxon test) or between DVF and BVF (*p* = 0.72, Wilcoxon test) (Figure 3).

The motor task did not modulate the SICF between experimental conditions (*χ* ^2^(2) = 2.4, *p* = 0.31; Friedman test) (Figure 3).

The % of change in MEPs, SICI, and SICF of ADM (task unrelated) muscle during FDI muscle activation did not show any significant differences between the 3 experimental conditions (*p* > 0.05 for all comparisons).

## 4. Discussion

The present study aimed at investigating MVF-related changes in ipsilateral motor cortex excitability and the intracortical inhibition/facilitation during a phasic unilateral motor task. The major finding was that the excitability of the ipsilateral corticospinal tract was increased when a visual feedback from the active hand was provided either directly or through a mirror but not when the visual feedback was blocked. In addition, MVF induced cortical disinhibition ipsilateral to the hand performing the motor task with respect to BVF. Moreover, these effects were restricted to the FDI homologue muscle contralateral to the active hand.

### 4.1. Changes in Motor Cortex Excitability

The performance of voluntary movements determines changes in corticospinal and intracortical excitability that are modulated differently depending on the hemispheric dominance and the unilateral or bilateral motor task execution [16]. Visual feedback of a hand performing a motor task seems to modulate both ipsilateral and contralateral M1 excitability and induces activation of cortical and subcortical areas that integrate the visual and proprioceptive input [11, 17, 18]. However, evidence for MVF-induced modulation of brain excitability is more controversial. In our study ipsilateral motor cortex excitability increased when visual feedback of the active hand was provided either directly or through the mirror, but not when vision of the hand was blocked, suggesting that visual feedback is of crucial importance of in motor cortex plasticity. However, MVF did not exert a more robust modulation of corticospinal excitability (as measured by MEP amplitude) compared with DVF condition, which is consistent with some previous reports [19, 20]. On the contrary, Garry et al. [21] and Carson and Ruddy [22] reported more pronounced increases in ipsilateral corticospinal excitability during MVF compared to direct vision of the active limb. We consider that the existing inconsistencies between the results of our study and previous publications are determined by differences in the applied methodology: some MVF studies evaluated the additive role of visual components to task-induced modulation of motor cortical excitability [20, 22]; others have used motor tasks synchronized to sounds [19]. Although, the differences between findings may be due to methodological details, additional experiments are required to explain the cause of the increase in MEP amplitudes. This could be due to reduced motor threshold, decreased intracortical inhibition, or increased intracortical facilitation [23]. Indeed, unilateral movements cause activity-dependent changes in MEPs of muscles on the other side of the body, evoked by stimulation of the hemisphere ipsilateral to the moving limb. These correlate with changes in ipsilateral SICI and force related changes in interhemispheric inhibition [24]. Moreover, unilateral voluntary movements can increase the silent period duration in the ipsilateral hemisphere (a marker of interhemispheric inhibition) without modulating MEP amplitude [25] suggesting that modulation of excitability of the corticospinal tract ipsilateral to a movement involves both local (intracortical) and remote (interhemispheric) modulatory mechanisms. These changes in the balance of excitation and inhibition of corticospinal neurons may help to select the population of cortical neurons responsible for the voluntary movement [26].

Cortical disinhibition is a relevant mechanism leading to motor recovery after stroke. Physiotherapy based on a “forced use” concept chronic stroke patients has revealed that motor cortical inhibition influences the reorganization pattern with treatment-associated cortical reorganization preferentially occurring in areas with reduced intracortical inhibitory properties that allows the cortical representation of the affected limb to expand in this direction [27]. Contrary to Reissig et al. [19], we observed a more robust cortical disinhibition (as measured by SICI) during motor task performance, specifically related to the MVF condition with respect to BVF, with no differences in modulation of SICF, which is in line with a previous study [18]. However, it should be noted that compared to our study, Reissig et al. [19] used a lower intensity for the conditioning TMS stimulus and a higher intensity for the test TMS stimulus in the SICI protocol at ISI of 3 ms that may account for differences with our findings.

The intracortical and interhemispheric inhibitory/excitatory changes have been shown to be dynamic activity-dependent processes [24] with SICI producing more global inhibition and similar effects on the transcallosal and descending corticospinal circuits; the SICF is thought to increase corticospinal output with no effect on interhemispheric inhibition [28, 29]. Our results show that changes in cortical excitability, intracortical inhibition, and facilitation of the ipsilateral hemisphere are limited to the area controlling the activity of homonymous muscle of the inactive hand, which is in line with previous studies [19, 30]. TMS studies suggest that selective activation of a hand muscle is accompanied by a selective suppression of intracortical inhibitory effects in the corticospinal neurons controlling that muscle [20, 31, 32] whereas motor imagery or observation of non-self-movements is associated with effector specific reduction of intracortical inhibitory circuits and an increased excitability of corticospinal tract [7, 33].

### 4.2. Visual Feedback in Motor Cortex Modulation

The major finding of this study was that the excitability of the ipsilateral corticospinal tract was increased when a visual feedback from the active hand was provided either directly or through a mirror but not when the visual feedback was blocked. In addition, MVF induced cortical disinhibition ipsilateral to the hand performing the motor task with respect to BVF.

MVF intervention in normal volunteers using a mirror box improved motor behavior and enhanced excitatory functions of the M1 after observation of a simple action, but not after repetitive motor training of the nontarget hand without MVF, pointing to the crucial role of visual feedback in cortical excitability modulation [34]. The motor cortex influences kinematic and dynamic parameters of movements, whereas the supplementary and primary motor areas use external or internal cues to trigger and guide movements [35]. The sensorimotor system controlling upper-limb movements may use both visual and proprioceptive inputs to formulate and calibrate motor commands in a synergistic fashion [36]. Indeed, MVF from the hand performing a motor task combined with passive movements applied to the inactive hand of the participant by an assistant produced greater increase in MEP amplitudes than from MVF alone [37]. Whereas visual feedback seems to be more important to induce movement illusion, proprioceptive/kinesthetic feedback is necessary to correct the illusion [38]. In experiments using the rubber hand illusion, synchronous visuotactile stimulation of a visible rubber hand together with one's own hidden hand elicits ownership experiences for the artificial limb. Varying the degree of synchrony between visual and tactile events by delaying tactile stimulation relative to visual feedback produced selective activation of the premotor cortex contralateral to the site of sensory stimulation reflecting the important role of premotor cortex in the integration of visual and somatosensory input [39]. The visual influence on ipsilateral motor cortex occurred even when proprioceptive input related to movement of the real opposite effector was incongruent with visual feedback of the hand given by the mirror.

Compared to a non-MVF versus MVF of a hand movement Matthys et al. [40] found 2 cortical areas uniquely associated with the mirror-induced visual illusion of hand movements: the ipsilateral superior temporal gyrus and the ipsilateral superior occipital gyrus. The superior temporal gyrus is a higher-order visual region involved in the analysis of biological stimuli and is activated by observation of motion [40] whereas the extrastriate body area (a region in the lateral occipital cortex) plays an important role in integrating different visual and sensory information determining the sense of ownership of the perceived body parts [41]. Indeed, the corticospinal facilitation is maximal when the observed motor task corresponds to the orientation of the observer [6] and is relevant to the MVF protocol to induce a more realistic perception of the illusory active hand compared to motor imagery or virtual reality feedback. Our study has some limitations: (1) the number of participants is small; (2) the order of experiment and MEP recordings was not counterbalanced; (3) we did not study the interhemispheric inhibition that could have played a role in ipsilateral cortical excitability changes.

## 5. Conclusions

Our findings indicate that visual feedback plays an important role in modulating motor cortex excitability but, compared to the BVF condition, MVF could present some advantages over DVF due to its more robust effects on cortical disinhibition. All together our results indicate that a combination of motor training with MVF therapy can induce significant neuroplastic changes through multisensory integration that is relevant to motor rehabilitation.

## Figures and Tables

**Table 1:** Demographic data of healthy subjects and rest motor threshold (RMT).

Subject	Dominant hand (R/L)	Sex (M/F)	Age (years)	RMT
1	R	M	36	48
2	R	F	29	44
3	R	F	30	62
4	R	F	29	54
5	R	F	28	50
6	R	M	32	50
7	L	F	26	44
8	L	F	27	44
9	R	M	27	49
10	R	F	21	42
11	R	M	41	32

R: right; L: left; M: male; F: female; RMT: rest motor threshold of nondominant; FDI: first dorsal interosseous muscle.

**Table 2:** Mean and standard deviation of MEP elicited by spTMS, SICI, and SICF in the nondominant FDI muscle at rest and during motor task of the dominant FDI muscle in different visual feedback.

Muscle	Experiment	MEP (mV)		SICI (mV)		SICF (mV)	
		Rest	Motor task	Rest	Motor task	Rest	Motor task
FDI	DVF	1.91 ± 1.50	2.63 ± 2.18^*∗*^	0.67 ± 0.82	0.73 ± 0.70	1.96 ± 0.90	2.82 ± 1.55^*∗*^
	MVF	2.03 ± 1.07	2.77 ± 1.38^*∗*^	0.75 ± 0.74	1.47 ± 1.47^*∗*^	2.66 ± 1.46	3.62 ± 2.01^*∗*^
	BVF	2.03 ± 0.78	2.14 ± 0.91	0.65 ± 0.50	0.77 ± 0.79	2.45 ± 1.12	2.95 ± 2.14

ADM	DVF	1.28 ± 1.40	1.16 ± 1.30	0.33 ± 0.31	0.33 ± 0.34	1.10 ± 0.89	1.17 ± 0.90
	MVF	1.36 ± 1.14	1.13 ± 0.95	0.65 ± 0.69	0.59 ± 0.64	2.79 ± 2.83	2.87 ± 2.90
	BVF	1.10 ± 0.93	1.20 ± 0.99	0.50 ± 0.53	0.51 ± 0.64	1.46 ± 1.16	1.51 ± 1.32

DVF: direct visual feedback; MVF: mirror visual feedback; BVF: blocked visual feedback. ADM: abductor digiti minimi muscle.

Wilcoxon test between motor task and resting state: ^*∗*^ *p* < 0.05.
